# From outsider to participant: a qualitative study about attaining and retaining work among persons with mental illness

**DOI:** 10.1186/s12889-024-20667-7

**Published:** 2024-11-30

**Authors:** Fredrik Stangeland, Vigdis Sveinsdottir, Eline Ree

**Affiliations:** 1 SHARE – Centre for Resilience in Healthcare, Faculty of Health Sciences, University of Stavanger, Stavanger, N-4036 Norway; 2 Health & Social Sciences, NORCE Norwegian Research Centre, Bergen, Norway

**Keywords:** Coping, Individual placement and support, Interviews, IPS, Mental health, Norway, Occupational rehabilitation, Qualification program, Self-efficacy, Supported employment, Workplace

## Abstract

**Background:**

In Norway, the number of people on sick leave and health-related absence from work has increased over the last few years, and mental illness is one of the main contributing factors. Individual Placement and Support (IPS) and the Qualification Program (QP) are two work-oriented measures offered by Norwegian labor and welfare authorities, aimed at increasing labor market participation among people with health-related problems. The aim of this study was to explore which factors people with mental illnesses found helpful for attaining and retaining work, and their experiences with taking part in IPS and the QP.

**Methods:**

The study had a qualitative explorative design. Eight semi-structured interviews with individuals who had attained and retained work after receiving IPS or taking part in the QP were conducted. The data were analyzed using systematic text condensation, a thematic and descriptive, cross-case analysis strategy.

**Results:**

The analysis revealed three main themes, each related to the contexts of work and daily life: (1) inclusion and support, (2) structure and routines, and (3) motivation and self-confidence. The participants emphasized the importance of feeling included and supported both at work and by their family and friends. Furthermore, structures and routines at work and in the participants’ daily lives kept them engaged and made them feel better, which was important for retaining work. Having the motivation to attain and retain work was essential and seemed to increase over time together with improved self-confidence as a result of being able to stay at work.

**Conclusion:**

Inclusion, support, structure, routines, and motivation are decisive for people with mental illnesses in changing their view of their health and increasing their independence and experiences of coping in work and everyday life. Taking part in work-oriented measures seemed to function as a “gateway” to changing behavior for the participants, both in work situations as well as more generally in life.

**Supplementary Information:**

The online version contains supplementary material available at 10.1186/s12889-024-20667-7.

## Background

Mental illness accounts for a large part of health-related absence from work in Norway, being the second largest diagnostic group for lost workdays due to sick leave [1] and the most common reason for disability benefits [2]. Within the context of mental illness, anxiety and depression symptoms/disorders are the most common diagnoses for sick leave while neurotic and behavioral disorders account for the majority in cases of disability. In addition to having vast economic consequences for society [3], being outside the labor market can negatively impact an individual’s mental and physical health [4, 5]. Based on the importance of labor market participation in promoting individual wellbeing and reducing socioeconomic costs, several measures have been implemented to help people attain and retain work. Work-oriented measures in Norway are organized by the Norwegian Labor and Welfare Administration (NAV) and are offered to people who need assistance to attain employment or to keep their job. In this study, we aimed to explore what individuals with mental illness who had received work-oriented measures in Norway experienced as important for attaining and retaining employment. We focus on two such measures: Individual Placement and Support (IPS) and the Qualification Program (QP) [6].

The purpose of the QP is to help people with reduced workability find their way into employment regardless of their diagnosis [7, 8]. The content of the QP is individually adapted based on the participants’ challenges, and each participant is followed up by a supervisor from the NAV. The support mainly consists of preparatory measures during the process of finding a job, but follow-up after employment may also be provided in some cases. Examples of activities in the program are motivational courses, work training/education, Norwegian classes, and financial advice [7, 9]. Previous research has shown promising results on work attainment after participation in the QP [10]. Other research and statistics from the NAV have also shown positive associations in the short term, but fewer of those who participated in the program retained work in the long term and many continued to receive various types of assistance from the NAV [11].

While the QP is a national program that has received limited attention in the research literature, IPS is an evidence-based intervention used in several countries aiming to help people with mental illness gain and keep employment [12]. While IPS was originally designed in the US for people with severe mental illness (e.g., schizophrenia), the intervention in Norway is offered to a broader patient group that also includes moderate mental illness and other challenges [13]. IPS is provided by an employment specialist following eight principles including a focus on finding ordinary paid employment as opposed to using preparatory measures or subsidized or unpaid work and individualized follow-up that continues for as long as each person wants and needs support, even after obtaining employment [12]. The intervention has shown good effects in 28 randomized controlled trials internationally [14] and has also been found to be effective in terms of employment and health-related outcomes in the Norwegian context [15].

Qualitative studies of people with mental illness who have received IPS in Austria, the Netherlands, Scotland, Sweden, and the US have found that motivation, regular follow-up, the employment specialist’s role, the participants’ skills and self-esteem, finances, and the working environment are experienced as important elements for attaining and retaining work [16–22]. The Norwegian labor market is characterized by an especially generous welfare system and high mental health-related unemployment gap compared to other countries [13], warranting investigations into which aspects of the support are considered crucial to aid work participation in this context. As of yet, there seem to be no published studies from the Norwegian context looking into how people with mental illness who attained employment through IPS experienced the intervention as helpful. Similarly, such qualitative research regarding participants’ experience of the QP is also lacking. Thus, while the effectiveness of interventions, particularly regarding IPS, is well documented [14], there is need for qualitative research to explore the experiences of participants who are successfully maintaining employment when receiving work-oriented measures, and which aspects of the support are considered helpful.

An important part of the work-related measures offered by the NAV is the idea that work has a positive impact on individual health and coping, and several studies have shown that self-efficacy has a positive impact on work participation [23, 24]. An individual’s self-efficacy is the belief in “*one’s capability to organize and execute the courses of action required to produce given attainments*” [25, p.3], and self-efficacy theory can provide a useful backdrop when exploring experiences of the process of finding and keeping employment.

The aim of this study was to explore what individuals with mental illnesses who had received IPS and participated in the QP experienced as important for attaining and retaining employment, focusing on the following research questions:

1. Which elements of the work-oriented measures from the NAV are perceived as useful in terms of attaining and retaining work?
2. What factors are experienced as important for participants managing to attain and retain work despite their mental illness?

## Methods

A qualitative explorative design [26] was chosen to explore individuals’ experiences of attaining and retaining work despite struggling with mental illness. A consent form, providing information about the purpose of the research, was distributed prior to the initiation of the study. Supervisors and IPS team leaders from the NAV recruited a purposive sample of participants who met the following inclusion criteria (see Table 1).

**Table 1:** Inclusion and exclusion criteria

Inclusion criteria	Exclusion criteria
age 18–50 years	< 18 years and > 50 years
mental illness duration > 3 months	mental illness duration < 3 months
maximum of two years since receiving IPS or participating in the QP	> two years since receiving IPS or participating in the QP
a minimum six months in regular paid work (part or full payment from the employer)	< six months in regular paid work (part or full payment from the employer) or vocational training
working 1–37.5 h per week.	Working < 1 h or > 37,5 h pr week

The sample included participants who lived and worked in urban and rural areas in the western part of Norway. All participants were in regularly paid part-time or full-time work. The participants’ occupational sectors included personal transportation, construction work, health, office and administration, retail, photography, and telecommunications. Six participants had received follow-up from job specialists providing IPS (a version of the intervention that did not include the IPS principle of collaboration with healthcare services) and two had received follow-up from supervisors in the QP. Sample size was guided by assessing the information power of the study [27], which refers to the depth, relevance, and quality of information obtained during the interviews, determining how much data is needed to reach a sufficient insight. The participants all had specific experiences relevant to the aim of the study and reported similar experiences with various nuances and perspectives. We consider the study’s information power to be high, as the sample was highly specific and relevant to the study aim and we received rich and specific data to illuminate the research questions of the study. The participants shared a variety of concrete experiences, the dialogues during the interviews were good, and the study was supported by theory, all of which are important dimensions for increasing the information power of a study [27].

Eight semi-structured individual face-to-face interviews were conducted between October 2022 and February 2023. Two participants withdrew without providing a reason. Seven interviews were conducted at a NAV office and one at the participant’s workplace. The interviews lasted from 35 to 55 min and were recorded using the Nettskjema application. Observational notes were taken during the interviews. A job specialist was present to support one of the participants who was unable to attend the interview independently. The job specialist did not play an active role in the interview. The main themes in the interview guide were how the participants managed to attain and retain work despite mental health problems (coping experiences), how their mental illness affected their workability, and which elements of the work-related measures they found helpful (psychological and social factors). The interview guide was pilot tested. The participants got the opportunity to comment on transcriptions and findings, but declined.

Systematic text condensation, a descriptive, cross-case analysis strategy, was used for the analysis [28, 29]. The analysis consisted of the following four steps: (1) reading through the data material to obtain an overall impression and establish preliminary themes, (2) developing code groups based on the preliminary themes and discussions between authors FS and ER and finding and sorting meaning units in the data material related to the code groups, (3) condensing the content of the code groups and establishing subgroups, and (4) synthesizing the condensates in each code group into reconceptualized descriptions of what individuals with mental illnesses experienced as important for attaining and retaining work. An overview of the analytical process is summarized in Table 2.

The authors have various professional experiences within clinical practice and research. FS has experience working with patients with mental health issues through various physiotherapy interventions and has experienced that factors beyond the intervention itself influence successful treatment and rehabilitation. ER and VS have backgrounds in health and work psychology. VS has considerable quantitative research experience within the fields of mental health and employment. ER also have quantitative and qualitative research experience in the fields of work and health, patient safety, organizational culture, patient involvement, and leadership.

**Table 2:** Excerpt of the analysis process using systematic text condensation

Preliminary themes	Code group	Subgroups	Condensate	Result section
Support Inclusion Self-awareness Routines Coping Finances Gradual work Independence	Inclusion and support in the context of work and daily life	Supportive collaboration with NAV Social support in daily life	Excerpt from the subgroup *Supportive collaboration with NAV*: *I can make suggestions and then we’ll figure it out together. The focus is on my capacities*,* what I can and can’t do. Diagnosis is a bit behind in a way and I get to decide for myself. I feel I am part of the process and it is in a way up to me how I want to run the race*	Excerpt from the subgroup *Supportive collaboration with NAV*: *(…) the significance of being seen as a human behind the diagnosis and being included in the work processes. The participants said that the focus of the employment specialist and supervisor was on their capacity and what they were able to do. Their diagnosis was unimportant*,* and participants experienced co-determination throughout the process*

## Findings

The analysis revealed several experiences and perspectives on why people with mental illness receiving IPS or the QP from the NAV managed to attain and retain work. The participants emphasized the importance of inclusion and support from the NAV and the people around them, structure and routines, motivation, and trusting themselves in their decision making. The following three main themes and sub-themes were developed:

1. Inclusion and support in the context of work and daily life.
  Work inclusion and supportive collaboration with NAV and employer.
  Social support in daily life.

2) Structure and routines in the context of work and daily life.
  Job routines provided structure and meaning in daily life.
  The importance of structure and routines for mental health.

3) Motivation and self-confidence in the work context and daily life.
  Motivation to maintain employment.
  The importance of work participation for self-confidence and coping.
  Changed mind-set when experiencing maintaining work.

The findings are elaborated below.

### Inclusion and support in the context of work and daily life

Inclusion and support from the supervisor and employment specialist at the NAV was described as important by the participants, as well as the significance of being seen as a human behind the diagnosis and being included in the work processes. The participants said that the focus of the employment specialist and supervisor was on their capacity and what they were able to do. Their diagnosis was unimportant, and participants experienced co-determination throughout the process. Being on the same “team” as the employment specialist and supervisor was considered essential for success. Other participants said the employment specialist and supervisor calmly explained how they could proceed independently to reach their goal and gave positive feedback and supported them throughout the process. The participants pointed out that they had to do their tasks such as job interviews themselves, but they knew the employment specialist would be there waiting for them afterward. Furthermore, they explained that it was of great value for them to have someone to contact when life was challenging. A participant said the employment specialist prioritized talking to her during an anxiety attack at work, even though the job specialist was at home. Some participants also pointed out the importance of getting an individual plan, understanding, and patience from their employment specialist and supervisor in order to gradually increase their ability to work. Flexibility from the employment specialist and supervisor was essential for maintaining work. A 25-year-old woman who had received IPS for several years said the following about her employment specialist’s approach:

*“It was just a focus that I should do this at my own pace. I should not feel any pressure on anything. It was as if you were respected for your goals*,* premises*,* and who you are*,* which made it much easier.”*

Inclusion and support from people other than the employment specialist and supervisor were also important for the participants. Participants emphasized their need to go out and socialize and admitted they often struggled to find someone who understood them and who they felt safe to talk with about their situation. Having someone to talk to who listened, understood, and gave advice was important. Some participants spoke about social life at work and how important it was to feel included, while others described severely challenging periods without work or social contact with friends that led to attempts at suicide. One participant described the lack of a “support system” around him outside of work as a crucial reason for why he could not previously attain work. Other participants pointed out the support of colleagues and increased social life at work as necessary. Good friends made the participants’ mental illness better. Some talked about a colorful and very inclusive work environment, while others emphasized that the support from friends and family helped them persevere. The participants talked about how it helped them mentally to go to work and meet others, especially on a bad day. A woman in her thirties who had managed to stay at work for more than four years emphasized the importance of support outside work: “*I have to try and reach out for support from others to get better somehow. It’s like “teamwork” in a way*,* but it’s hard to explain. I need support from people at home and from family and friends too.” *

### Structure and routines in the context of work and daily life

Taking part in society and enjoying routines was essential for the participants. It was necessary to have a plan, if not the day could be ruined. The participants described how their mood was affected by a lack of routines or structure in everyday life. They pointed out that having demands at work and in their daily life was important. Routines at work led to better routines and structure in their daily life. Job routines enhanced quality of life and gave a sense of mastery while at the same time creating new opportunities further on in life. They found it extremely good for their self-esteem to contribute to something. A job gave the participants input from society, and they became socially interactive. Without employment, they experienced a form of existence on the outside of society. A male participant pointed out the importance of being perceived like everyone else, like “normal people”, people who are employed. Participants talked about feeling important to themselves and others. One of the female participants emphasized the importance of enjoyable routines:

*“Routines are fundamental*,* but it must be routines you enjoy. Because when I was at my previous workplace*,* I could not do it. I pushed myself further down into a ‘pit’ and I could not escape for a long time. Because I was uncomfortable*,* I could not make it work for me.”*

Routines affected the participants’ mental state, and the participants described their mental illness as much worse outside employment. Their mental state did not improve without routines, and the participants talked about loneliness and a poor sense of self when they were not employed. They expressed frustrations when they were unemployed and constantly thought negatively about themselves. One participant said she became more depressed without a job, and others pointed out that they had more negative thoughts, significant mood swings, and anxiety when unemployed. The participants felt better when being able to go to work rather than just staying at home. Some participants said they “forgot” their mental illness when they went to work and stated that life had improved when they started working and meeting other people. A 26-year-old woman who had been working full-time for 1.5 years said: *“Without a job and routines*,* you have a lot of spare time and will start overthinking. Then you will not get any better and you go grinding and pushing yourself further down.”*

### Motivation and self-confidence in the work context and daily life

The participants worked because they believed they had something to contribute with, and having a place to contribute was necessary for the participants’ mental health. Some stated that the feeling of being helpful was decisive, while others said they could motivate themselves for work because it was meaningful and exciting. One man pointed out the difficulty of maintaining motivation if work was only about payment, while others emphasized that a permanent job and follow-up from the NAV made life easier because they had a reasonable income and did not have to go to charities to ask for food and support. The participants said the job gave them a spark of life and a sense of mastery and made them more independent. The motivation they got from working with something enjoyable was expressed as follows by one of the participants who had been at the same workplace for more than three years: *“There is something in me that means I cannot focus on what I am doing if I am not doing something I like. I look forward to going to work every single day.”*

All participants pointed out that the sense of being independent at work and in everyday life strongly contributed to them managing to retain work. One participant said she felt a sense of mastery, considering that she conducted the job interview all by herself and therefore deserved the job. There was no one else who did the talking for her. Other participants talked about freedom of choice, being their own boss, and managing their working days. The participants said they were more independent at work and in daily life after receiving IPS and participating in the QP because they had gained self-confidence. If they failed, they tried again and did not avoid difficult tasks like they would have before. Due to the work-oriented measures and their supervisor or employment specialist, the participants found their strengths and what suited them instead of just being hired for a position, something they had not experienced in the ordinary labor market previously. A male participant went to upper secondary school in another city when he was on disability benefits. He saw other students succeed at school and later at work, which motivated him and increased his self-confidence to finish school and further master employment. The following quote from one of the participants illustrates the role of IPS in helping the participant cope: “*Out of the blue*,* I’ve started handling tasks better. Before IPS*,* I used to shy away from challenging tasks*,* but now I’m up for trying anything*,* and it usually turns out great”.*

Participants said that it required much self-insight and self-improvement to figure out what they could do over an extended period. One woman emphasized that she could now see the positive things; for example, when people gave feedback at work, she knew it was because they wanted her to improve and were not trying to be mean. The participants pointed out a change from believing they could not work to thinking they were able to retain work. Their mental illness was still there, but through increased skills at work and in daily life they found a reason for staying at work. Participants expressed that they now were able to identify their psychological stressors earlier when they arose. After participating in IPS or the QP and working, they were less reluctant to use health and social services such as psychologists, GPs, physical therapy, and the NAV. Some participants said they were misdiagnosed and incorrectly medicated from their youth, and when they finally got the correct diagnosis and medication it was as if a “cloud” had disappeared from their mind. Without this cloud, it was easier to master work and life in general, as illustrated by this quote from a 25-year-old woman: “*I`m not letting those negative thoughts hold me back like I used to before IPS. I’ve changed my mindset and now I focus on the positive side of socializing at work and in everyday life”.*

## Discussion

**Fig. 1:**
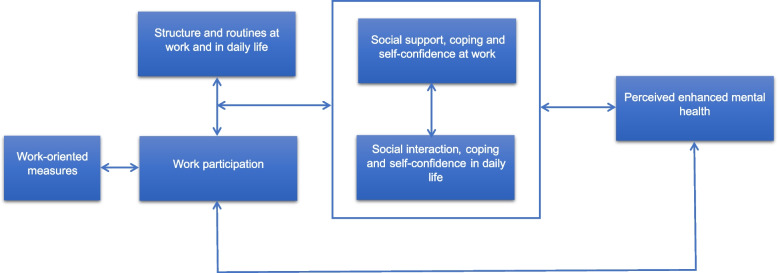
Conceptual map of the relationship between work participation and perceived mental health

The findings presented here demonstrate the importance of inclusion and social support in the context of work and in personal life as well as the importance of structure and routines, motivation, and self-confidence for people with mental illness in attaining and retaining work in Norway. The participants experienced that these factors contributed to changing their self-awareness, independence, and mastery experiences, enabling them to cope with staying at work. The findings can be illustrated in a conceptual map (Fig. 1), showing the interdependence between participants’ experiences at work and in their daily lives. More specifically, the social support, structure and routines encountered at work contributed to structure, social interactions and a sense of meaning in the participants’ daily life. These aspects had a positive impact on the participants’ coping and self-confidence, improving their mental health, which in turn contributed to retain work.

Our study is in line with previous qualitative studies on IPS, showing that participants experienced motivation, regular follow-up, the role of the employment specialist, and the participants’ own skills and self-esteem as important elements to attaining and retaining work [16–22]. Our study adds to the associations between these factors, the importance of good collaboration between the participant and employment specialists, and how essential the participants’ own contributions are for staying at work in the long term. Existing research regarding the QP has mainly addressed the service providers’ experiences of the program [7, 8]. One previous study has examined participants’ experiences and perspectives of the QP while participating in the program [30], however, without focusing on factors experienced as important for attaining or retaining work. As the first to explore these perspectives, the current study illustrates which aspects are perceived as especially important among participants receiving work-oriented support in the Norwegian context.

In general, our findings resonate with classical descriptions of beneficial factors that may contribute to explaining the health-promoting influence of work on the individual, including time structure, social contact, collective purpose, status, and activity [31]. This seems especially pertinent regarding the influence of the social aspect in participants’ descriptions of their processes before and throughout the return to work, including previous lack of social support, relationships with the support person in the intervention, contact with colleagues, and expanded social connections. The importance of social support for the participants’ ability to cope with staying at work may be explained through the self-efficacy theory [25], which emphasizes the importance of having a trusting social network, such as family and relatives, friends, or colleagues, for coping with challenging situations. The findings indicated that the participants’ experience of coping may have increased both by observing other employees who received work-related measures succeed with staying at work and by their own experiences of managing to attain and retain work despite their mental illness. This is in line with previous studies, where inclusion and social support have been documented as essential for the ability of people with mental illness to retain work [16–19]. Lima and Furuberg [7] found promising results for the QP in areas with low unemployment, which considering Banduras’ theory of self-efficacy may be due to observation of peers at work because vicarious experiences are one of the primary resources for self-efficacy [25, 32, 33].

Through IPS, the QP, and by being at work, it appears that our participants have built social networks that they trust. Coombes et al. [20] found that the role of the employment specialist changed over time for participants receiving IPS, where the support and external motivation from the job specialist was perceived as more important in the beginning. The participants in our study reported similar experiences, as the employment specialist’s role was very important in the beginning when the participants had low individual and social manageability but seemed to become less important over time. This can probably be explained by the participants expanding their social networks and becoming more independent at work and in daily life through experiences of mastery, which is the most important factor in increasing self-efficacy [25, 32, 33]. However, Raeside & McQueen [21], who interviewed participants receiving IPS in Scotland, emphasize that continuous follow-up by the employment specialist (or occupational therapist), if the participant needs it, is crucial for staying at work. Participants in the current study pointed out that having a person to call when needed made them feel safe. This may illustrate the need for a continuous and close social network surrounding the participants to help them manage to retain work.

The feeling of inclusion and contributing to society has been shown to be significant for staying in employment [16]. Similar to the findings in the study by Raeside & McQueen [21], IPS and the QP seemed to help several participants in the current study create good routines in their daily life outside of work. By being active, participating, and feeling a sense of belonging, the participants experienced that their mental health improved and helped them to stay employed. These findings are supported by Donovan et al. [34] who point out that activity, belonging, and commitment promote mental health.

The flexibility and individual adaptation of working hours and tasks was perceived as crucial for the participants’ ability to stay at work. This flexibility may be why the participants’ routines did not increase stress levels and reduce energy, as Koletsi et al. [18] and Areberg et al. [19] found in their research, but rather the opposite. Understanding work as meaningful and having substantial motivation increased the participants’ effort and commitment to change their self-perception and to search for social networks and support in people around them, thus becoming more independent in daily life and the work context. Motivation, increased self-confidence, coping with psychological symptoms, and social networks have previously been shown to be essential for attaining and retaining work through IPS [16, 17, 19].

The findings of the current study show different individual experiences of how and why people who received IPS and participated in the QP managed to attain and retain work, indicating that there is no work-orientated measure that fits everyone. Nevertheless, it seems that the work-oriented measures may have functioned as a “gateway” to changing behavior, both in work situations and in daily life. By being shown understanding and support for their challenges from the start, being seen as a human being “behind the diagnosis”, and preparing an individually adapted return to work, the participants gained positive coping experiences, increased their social networks, and received verbal feedback from employment specialists, supervisors, colleagues, and friends. The verbal feedback was consistent with the mastery experience and appears to have changed the participants’ cognitive understanding, self-confidence, and independence. In summary, the participants received all four primary sources of coping strategies – including mastery experiences, vicarious experiences, verbal persuasion, and physiological and affective state [25, 32, 33] – which might help explain improved functioning in the work context as well as in everyday life.

## Strengths and limitations

The main strength of this study is the access to different perspectives on what helped our participants with mental illness attain and retain work. This is a challenging group to recruit to qualitative studies, and it requires effort to make participants feel safe enough to talk openly about their experiences. The main limitation of this study is the relatively small sample, which does not allow for generalization of the findings or specific conclusions about the two work-oriented measures of IPS and the QP. However, this study was a qualitative exploration of what the participants found helpful in attaining and retaining work and was not an evaluation of IPS and the QP, thus making information power and trustworthiness [27] more relevant than sample size. As detailed in the methods section, our sample provided sufficient data to explore our study aim.

This is the first study to our knowledge that explores the experiences of attaining and retaining work among individuals with mental illness who have received IPS and participated in the QP in a Norwegian setting. The participants were a selected group who had all received work-oriented measures from the NAV and succeeded in obtaining work prior to being included in this study. Six of the participants received IPS, which requires that the participants are motivated to start or maintain work [35]. People who are motivated and still manage to stay at work may have other experiences than those with less motivation and who are unemployed. The participants participating in the QP cannot receive disability benefits or work assessment allowance [7, 9], which means that these participants are also a selected group that may in some respects have better opportunities to succeed in work. Therefore, unsuccessful experiences with obtaining or retaining work through IPS or the QP are not highlighted and fall outside the focus of this study.

The first author (FS) is health professional with limited experience in conducting interviews. However, his knowledge of the field and experience with the patient group was considered a strength to ensure good dialogues during the interviews, which is crucial for achieving high information power [27, 29]. FS is employed at NAV, although not in a role related to work-oriented measures, which could potentially introduce biases related to theoretical perspectives and anticipated outcomes. However, the other authors (VS and ER) work as researchers and have extensive research experience, including on IPS (VS), ensuring a sound research process with a high degree of reflexivity and transparency [28]. The use of a systematic analysis method and attention to reflexivity helped ensure that the study was not an evaluation of IPS or QP, but rather participants were encouraged to discuss their work experiences irrespective of the work-oriented measures they had received.

Although the study involved individuals with persistent mental illness, the findings might be transferable to work-oriented measures for other diagnostic groups, given that coping with illness is inherently subjective regardless of the diagnosis. The knowledge gained from this study can be useful for employers in facilitating for individuals with chronic conditions to maintain or retain employment.

## Conclusion

This study provides insight into the experiences of people with mental illness who have succeeded in attaining and retaining work through work-oriented measures in Norway. The findings highlight how social support, structures, routines, and motivation changed the participants’ understanding of their self-awareness, independence, and mastery experiences, enabling them to attain and retain work and master their daily lives. The participants went through a gradual process from being outsiders to being participants in the ordinary labor market and being working members of society. The work-oriented measures seem to have functioned as a “gateway” to help the participants start these lifestyle changes. While this study focused on individuals with persistent mental illness receiving two specific work-oriented measures, coping with illness is subjective regardless of the type of diagnosis, and these experiences can thus also be relevant across other vulnerable groups receiving assistance to enter working life. We propose that individualized support and a focus on the social inclusion of individuals struggling with attaining and retaining work can be important keys to success in the process of recovery and vocational rehabilitation of people with mental illness. Knowledge about factors contributing to successful employment among individuals with mental health problems has important implications for individual health and wellbeing as well as in a broader societal perspective.

## Supplementary Information

Supplementary Material 1.

## Data Availability

Due to the requirements set by the Data Protection Official for Research (NSD), we are only allowed to share the summarized and analyzed data and not the transcripts or other data material that might identify the participants. Anonymized data material can be made available upon reasonable request to the corresponding author of this manuscript. Data was collected using the Nettskjema dictaphone application.
