# Supplementary material for: From outsider to participant: a qualitative study about attaining and retaining work among persons with mental illness
Source: BMC Public Health. 2024 Nov 30;24:3346. doi: 10.1186/s12889-024-20667-7 (PMC11608475; doi:10.1186/s12889-024-20667-7)
Supplement: Supplementary file 1 — Supplementary Material 1. [file 12889_2024_20667_MOESM1_ESM.docx]

**Interview Guide**

**Introduction**

Name

Age

Occupation (previous and current)

Family

**Opening Questions**

- Can you describe how you discovered work-oriented measures (WOM) and how you proceeded to make contact?

- How was your first experience with WOM?

- What did you perceive to be the focus of NAV during your first meeting with WOM?

**Main Questions:**

*How do you manage to stay employed?* (Regardless of WOM.)

- What resources do you consider crucial for your ability to remain employed? (e.g., support from NAV, salary, support from family/friends/colleagues/supervisors, independence). In what ways, could you elaborate?

- What have you personally done/do to cope with staying employed?

*Which aspects of WOM have been perceived as useful/less useful in terms of maintaining employment?*

- Can you provide examples/elaborate? Was there a particular experience or event that changed your perception?

- How do you experience that AVT has influenced you in terms of staying employed?

- What differences do you experience in staying employed before and after WOM?

- Which factors/elements do you perceive WOM emphasizes that have had/have significance for you? In what ways, can you provide examples?

*How does mental illness affect the possibilities of staying employed?*

- In what ways do you experience that your mental illness affects your daily life?

- How has your experience of mental illness been when you have been unemployed compared to when you have been employed?

**Wrap-up:**

What advice would you give to the NAV regarding WOM? What changes would you make to improve WOM for yourself? What changes could NAV make to improve WOM further?

Finally, could you say something about how it has been to participate in this interview?
